# Multi-view 3D skin feature recognition and localization for patient tracking in spinal surgery applications

**DOI:** 10.1186/s12938-020-00843-7

**Published:** 2021-01-07

**Authors:** Francesca Manni, Marco Mamprin, Ronald Holthuizen, Caifeng Shan, Gustav Burström, Adrian Elmi-Terander, Erik Edström, Svitlana Zinger, Peter H. N. de With

**Affiliations:** 1 Department of Electrical Engineering, Eindhoven University of Technology, Eindhoven, The Netherlands; 2 Philips Healthcare, Best, The Netherlands; 3 Shandong University of Science and Technology, Qingdao, China; 4 Department of Neurosurgery, Karolinska University Hospital and Department of Clinical Neuroscience, Karolinska Institute, Stockholm, Sweden

**Keywords:** Patient tracking, Spinal surgery, Skin tracking, Surgical guidance, Feature localization

## Abstract

**Background:**

Minimally invasive spine surgery is dependent on accurate navigation. Computer-assisted navigation is increasingly used in minimally invasive surgery (MIS), but current solutions require the use of reference markers in the surgical field for both patient and instruments tracking.

**Purpose:**

To improve reliability and facilitate clinical workflow, this study proposes a new marker-free tracking framework based on skin feature recognition.

**Methods:**

Maximally Stable Extremal Regions (MSER) and Speeded Up Robust Feature (SURF) algorithms are applied for skin feature detection. The proposed tracking framework is based on a multi-camera setup for obtaining multi-view acquisitions of the surgical area. Features can then be accurately detected using MSER and SURF and afterward localized by triangulation. The triangulation error is used for assessing the localization quality in 3D.

**Results:**

The framework was tested on a cadaver dataset and in eight clinical cases. The detected features for the entire patient datasets were found to have an overall triangulation error of 0.207 mm for MSER and 0.204 mm for SURF. The localization accuracy was compared to a system with conventional markers, serving as a ground truth. An average accuracy of 0.627 and 0.622 mm was achieved for MSER and SURF, respectively.

**Conclusions:**

This study demonstrates that skin feature localization for patient tracking in a surgical setting is feasible. The technology shows promising results in terms of detected features and localization accuracy. In the future, the framework may be further improved by exploiting extended feature processing using modern optical imaging techniques for clinical applications where patient tracking is crucial.

## Background

The insertion of pedicle screws is a critical step in spine fixation surgery. Conventional open surgery is performed through a mid-line incision where the posterior aspect of the spine is exposed. However, there is a trend toward increased use of minimally invasive surgical techniques, due to reductions in blood loss, length of hospital stay, and surgical site infections [1]. Minimally invasive surgery (MIS) is performed through small skin incisions where the vertebrae are reached by use of tubular retractors [2]. Due to the reduced visibility during MIS procedures, intraoperative imaging such as fluoroscopy is frequently used. However, to reduce radiation exposure and increase accuracy, a number of computer-assisted navigation solutions have been devised [3–6]. Clinical studies have shown that the use of intraoperative three-dimensional (3D) imaging coupled to a navigation system leads to higher accuracies than competing technologies [7]. All navigation technologies require co-registration of the patient and the pre- or intraoperative images to allow tracking of both patient and surgical instruments relative to the medical images. Conventional navigation solutions typically include infra-red camera systems tracking a dynamic reference frame attached to a vertebra [8]. Efforts have been made to design patient tracking methods based on unobtrusive markers or no markers at all. One such system using non-invasive optical markers has been described by Malham et al. [9, 10] (SpineMask, Stryker, Kalamazoo, Michigan, USA). The system enables high accuracy placement of minimally invasive lumbar pedicle screws. Markerless tracking solutions have been used experimentally on phantoms in other surgical fields. However, studies on spine surgery are lacking [11, 12]. A robot system using light to track the bony anatomy and performing pedicle screw placements was recently presented. The device was validated on cervical vertebrae phantoms, reaching a mean positional error of 0.28 ± 0.16 mm [13]. The Microsoft Hololens uses surface matching for tracking and has been used experimentally in non-medical phantoms with an accuracy ranging from 9 to  45 mm [14], while in a spine phantom study, an accuracy of roughly 5 mm was achieved [15]. The navigation technology used in this study is an augmented-reality surgical navigation (ARSN) system relying on adhesive optical skin markers for motion tracking and compensation [16, 17]. Four high-resolution optical cameras are integrated in the flat detector of a C-arm with cone-beam computed tomography (CBCT) capability. The markers are recognized by the cameras and their relative positions in space are used to create a virtual reference grid, which is co-registered to the patient during CBCT acquisition. Optical markers attached to the patient’s skin have been used for respiratory motion tracking [18] and for medical imaging applications [19]. The use of optical markers for motion tracking is combined with digital image correlation and tracking techniques [20–23]. Recently, Xue et al. [24] demonstrated that ink dots on the skin could be video tracked with high precision and that the post-processing retrieved more detailed information compared to marker-based methods. Similarly, direct tracking of spine features and tracking of skin features using hyperspectral cameras for spine surgery have recently been proven feasible.

In this study, a new markerless tracking technology using grey-scale video cameras based on skin feature detection was evaluated. Image analysis techniques were applied to detect and track natural features of the skin. There are several advantages when refraining from using optical markers for motion tracking. First, the workflow of the procedure can be improved by simplifying the protocol for patient preparation and by increasing the reliability of tracking during the surgical procedure. Second, the risk of losing sight of the markers can be abolished when skin features can be used as a reference. The well-known feature detection algorithms, Maximally Stable Extremal Regions (MSER), and Speeded Up Robust Features (SURF) were applied to detect and extract skin features such as moles and pigment spots. These methods were chosen, since they offer a good reproducibility under different image views, being invariant to rotation, scaling, and affine transformation [25–28]. The proposed 3D-localization framework, used multi-view geometry principles to perform image rectification, enhance feature detectability, improve feature matching, and calculate and assess each triangulated feature. The sum of squared differences (SSD) was used as a feature matching metric on scan lines between multiple-view acquisitions. To remove 3D outliers, a second feature selection step was applied, specifically performed for the z-coordinate mismatch after the triangulation of all pairs of matched features. The final inliers were evaluated by computing the overall mean triangulation error. In summary, the contribution of this paper is an alternative to marker-based tracking. We hypothesize that the camera feed provides enough details for skin feature detection and tracking. The sub-millimeter localization accuracy achieved was sufficient for surgical navigation. The framework included 3D reconstruction and feature localization over multiple-view acquisitions. It was validated on eight clinical spinal surgery cases performed in an academic tertiary medical center. This paper concentrates on the application of skin feature detection techniques to achieve accurate markerless tracking in spinal surgery. In the development of tracking systems, feature detectors and descriptors are widely investigated, since they demand the highest percentage of the processing time. The former is dependent on available image information, while the latter defines the encoding [29]. Aspects such as adaptability to image transformation and mismatched features need to be evaluated, as they can potentially affect tracking [30].

In this paper, we evaluate different feature detectors and extractors (SURF and MSER), for studying the number of inliers and the overall localization error on multi-view images from several spine patients, subjected to different illumination conditions. In addition, to strengthen the stability in tracking by eliminating the mismatched multi-view keypoints and improve image matching, we proposed a 3D outlier removal step, imposing the matching to the keypoint relying on the same epipolar line. The 3D triangulations were obtained only from the matches relying on the epipolar constraint. The contributions are: (1) building a computer vision framework for preprocessing optical skin images, detecting and matching local invariant image regions for two different image views; (2) assessing the most stable feature detection approach for reliability and accuracy; (3) improving the image matching by introducing a 3D epipolar constraint; (4) validating the methodology on optical images acquired in eight patients to assess the 3D-localization error for the matched features.

## Results

**Fig. 1:**
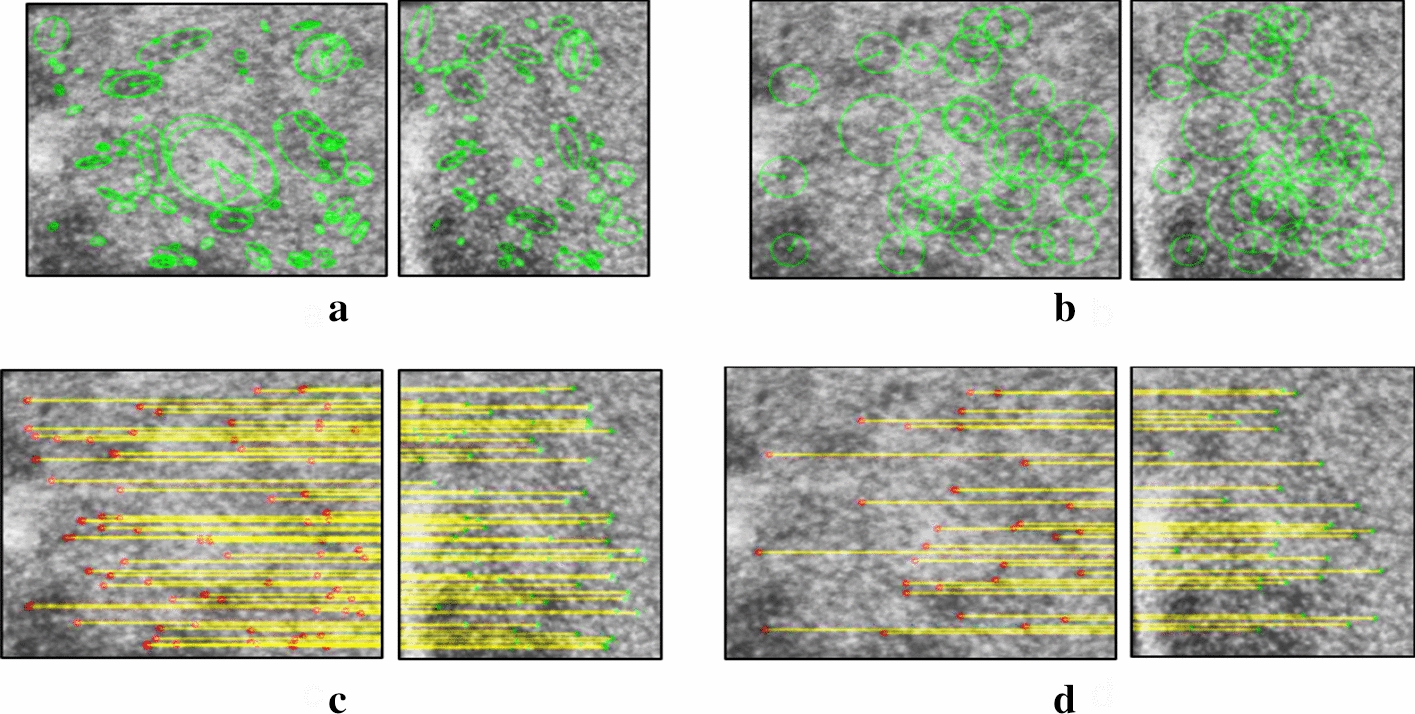
Two examples of MSER and SURF feature detections (**a**, **b**) and corresponding matches (**c**, **d**) at the same location for an image pair

Optical data for assessing the 3D localization were collected from two sources: a cadaver study and a prospective clinical observational study. The cadaver study was performed according to all applicable laws and directives. The clinical study was approved by the local ethics committee and all enrolled patients signed informed consent. The data that support the findings of this study are generated by Philips Electronics B.V., Best, The Netherlands and Karolinska University Hospital, Stockholm, Sweden. All images of the datasets were acquired at the same UHD resolution of 2592 pixels by 1920 lines. The first dataset consisted of one multi-view acquisition, thus four images, of a cadaver. MSER and SURF were applied to several selected regions to perform the first multi-view experiment for skin localization. The second dataset consisted of image data from eight patients included in a spine navigation study and taken during the surgical procedures. The data were used to perform two different experiments, first a skin feature localization and later an optical marker localization and the ground-truth comparison. In Table 1, the total number of analyzed frames during the acquisitions and the corresponding acquisition times are reported. All patients were classified by the physicians regarding Fitzpatrick Skin Type I, II, or III. The first feasibility study was performed by analyzing the skin of the cadaver. The localization system was used for four flat selected regions with the c1|c3 camera pair. The features detected for this dataset were triangulated with a mean triangulation error of 0.239 mm for MSER and 0.218 mm for SURF. Due to its intrinsic functional operation, the MSER algorithm detects multiple blobs located at the same coordinates. This explains why MSER seems to have the capability to detect more features than SURF. The discarded-feature ratio of the matched features to the selected inliers was 3.96 ± 0.80 and 2.93 ± 0.45 for MSER and SURF, respectively. The clinical dataset involved patients undergoing open surgical procedures via mid-line incisions along the spine. Several plane regions were carefully selected for some patients (2nd, 4th, and 7th), where the skin was partially covered by blood. The performance results of the localization framework for all the eight patients in the study are shown in Tables 2 and 3. Descriptive statistics for triangulation error for each detection method are reported in Tables 2 and 3. Figure 1 shows two examples of MSER and SURF feature detection and corresponding matches at the same location for an image pair.

**Table 1:** Patient data acquisitions

Patient no.	Acquired images	Acquisition time (hh:mm)	Starting time	Ending time
1	188	3:21	12:20	15:41
2	424	4:41	10:32	15:13
3	204	2:20	10:55	13:15
4	112	3:18	10:20	13:38
5	132	5:22	9:46	15:08
6	160	4:35	11:20	15:55
7	284	3:40	10:55	14:35
8	12	0:03	13:07	13:10

**Table 2:** Triangulation error analysis—MSER

	MSER								
Patient no.	1	2	3	4	5	6	7	8	TOT
Analyzed regions	1	1	1	7	11	3	7	3	ALL
Area [$\hbox {cm}^2$]	57.8	18.6	27.6	183.9	232.8	75.5	80.0	29.5	705.6
Number of features	366	125	60	1372	1511	264	1083	153	4934
Mean [mm]	0.167	0.162	0.176	0.191	0.269	0.164	0.170	0.214	0.207
Std deviation [mm]	0.110	0.088	0.099	0.122	0.159	0.109	0.123	0.128	0.139
Features/$\hbox {cm}^2$	6.3	6.7	2.2	7.5	6.5	3.5	13.5	5.2	7.0
Rms [mm]	0.200	0.184	0.202	0.226	0.313	0.197	0.210	0.249	0.249
Min [m]	0.377	3.712	1.252	0.328	0.009	0.251	0.008	5.230	0.008
Quartile 1 [mm]	0.068	0.106	0.103	0.091	0.136	0.066	0.068	0.108	0.091
Median [mm]	0.164	0.160	0.188	0.181	0.267	0.150	0.149	0.190	0.190
Quartile 3 [mm]	0.244	0.218	0.241	0.276	0.389	0.242	0.247	0.314	0.303
Max [mm]	0.448	0.373	0.427	0.537	0.649	0.422	0.593	0.518	0.649

**Table 3:** Triangulation error analysis—SURF

	SURF								
Patient no.	1	2	3	4	5	6	7	8	TOT
Analyzed regions	1	1	1	7	11	3	7	3	ALL
Area [$\hbox {cm}^2$]	45.2	18.9	29.1	145.5	157.4	48.0	49.8	20.7	514.7
Number of features	130	78	42	577	457	83	299	61	1727
Mean [mm]	0.184	0.148	0.126	0.199	0.268	0.172	0.160	0.195	0.204
Std deviation [mm]	0.113	0.091	0.103	0.125	0.148	0.110	0.120	0.117	0.134
Features/$\hbox {cm}^2$	2.9	4.1	1.4	4.0	2.9	1.7	6.0	2.9	3.4
Rms [mm]	0.216	0.174	0.163	0.235	0.306	0.204	0.200	0.228	0.244
Min [m]	1.644	1.944	1.235	0.658	1.782	1.153	0.075	2.542	0.075
Quartile 1 [mm]	0.091	0.066	0.037	0.102	0.151	0.073	0.060	0.109	0.090
Median [mm]	0.193	0.150	0.121	0.194	0.272	0.178	0.140	0.174	0.195
Quartile 3 [mm]	0.270	0.219	0.185	0.286	0.376	0.245	0.237	0.265	0.300
Max [mm]	0.427	0.340	0.399	0.543	0.620	0.433	0.575	0.486	0.620

**Fig. 2:**
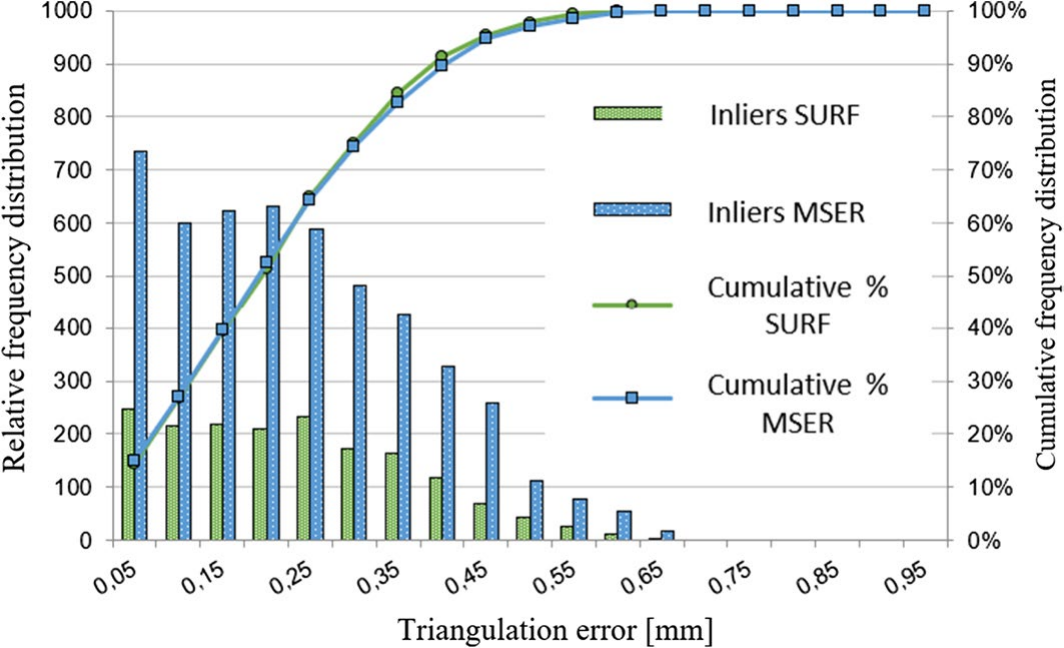
Triangulation error distribution (MSER and SURF)—patient data

**Fig. 3:**
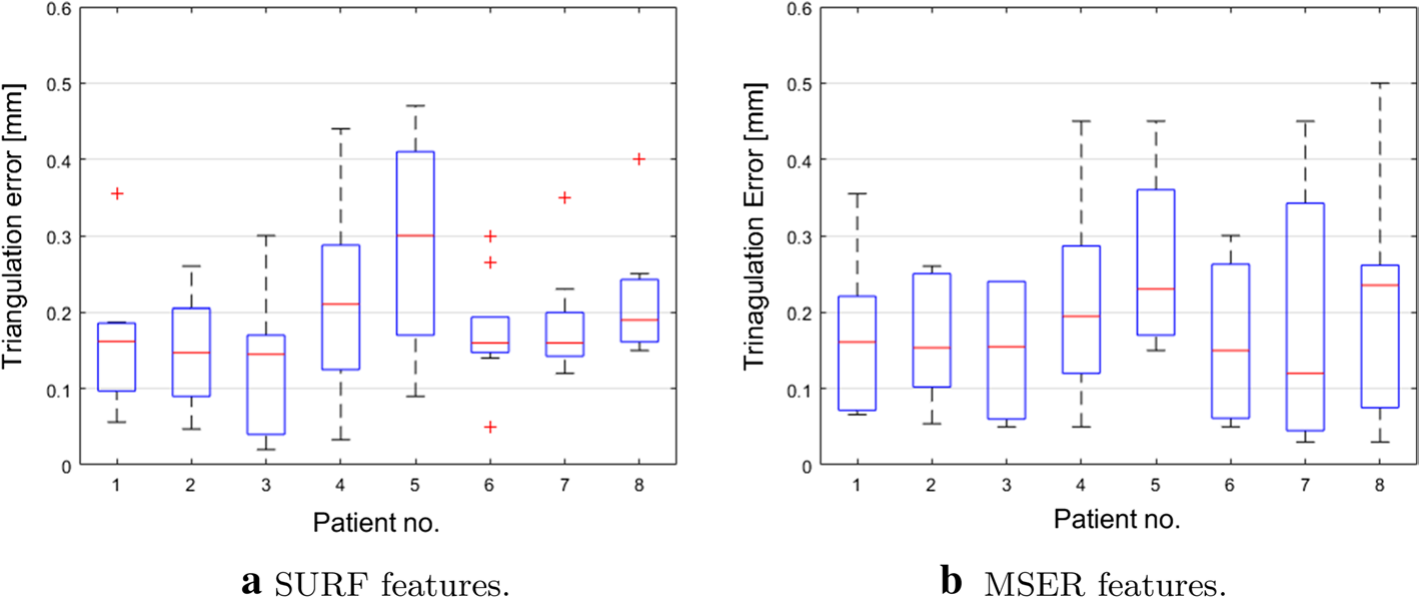
Boxplots for the mean triangulation error in eight patients. The red line represents the median. Upper and lower limits depict the 75th and 25th percentiles, respectively. The min and max values are visualized with whiskers, and the outliers are shown with ’+’ symbol

**Fig. 4:**
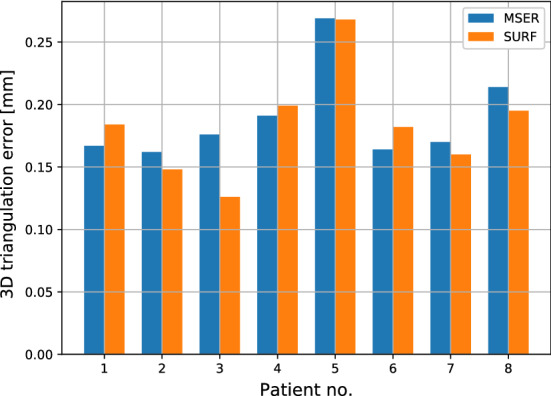
Bar plot visualizing the triangulation error (MSER and SURF)

On a total amount of 4934 (MSER) and 1727 (SURF) features, mean triangulation errors of 0.207 and 0.204 mm were reached for MSER and SURF, respectively. An important observation was that 75% of the detected features had a triangulation error within 0.3 mm (Fig. 2), appropriate for spinal surgery applications. The discarding ratio of the matched features to the selected inliers in this case was 3.73 ± 2.69 and 2.61 ± 1.70 for MSER and SURF, respectively. The median errors show a similar variability in the triangulation error when SURF and MSER are used, respectively (Fig. 3a, b). The variability and the outliers may be caused by lighting differences or limited visibility of the skin area [31]. The triangulation error using SURF and MSER for each individual case is depicted in Fig. 4.

Two-sample t tests were used to assess differences when using MSER and SURF. A p value of less than 0.05 was considered statistically significant. No statistically significant differences between the two methods were found (p > 0.05). A two-sample t test was also performed to assess the accuracy of the markerless approach, which was found to be superior (p < 0.05) compared to the ground truth (marker-based detection). A significant statistical difference was also found when detecting skin features among different patients (p < 0.001). This can reflect differences in the number of analyzed frames per patient, illumination conditions, number of detected features, and skin type. In this case, the f test rejects the null hypothesis at the default 5% significance level and suggests that the true variance is greater than 25%.

**Fig. 5:**
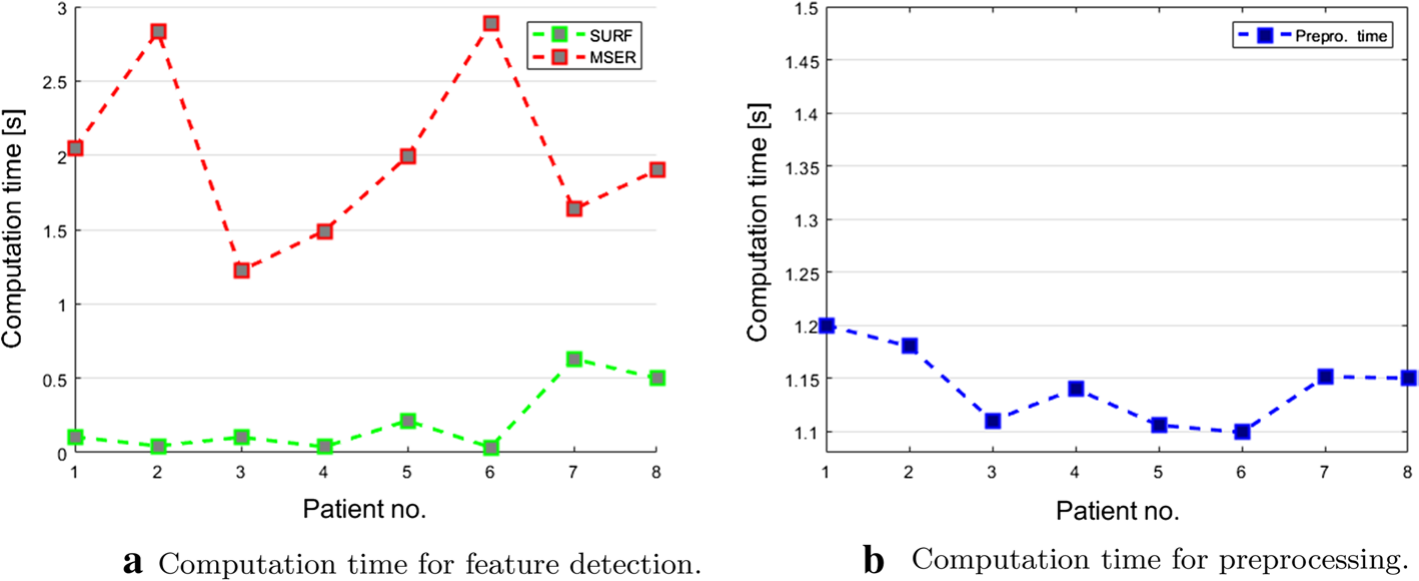
Computation time for the preprocessing and skin feature detection

The computation time for the skin feature detection was on average 0.19 and 1.86 s per frame when SURF and MSER were used respectively. Per-patient results are visualized in Fig. 5a. With a mean of 5 fps, SURF is most suitable for a future real-time implementation. The preprocessing step reached a computation time of 1.14 s. Notably, for real-time navigation, shortened preprocessing times may be achieved with improved lighting conditions.

### Marker localization and ground-truth comparison

**Fig. 6:**
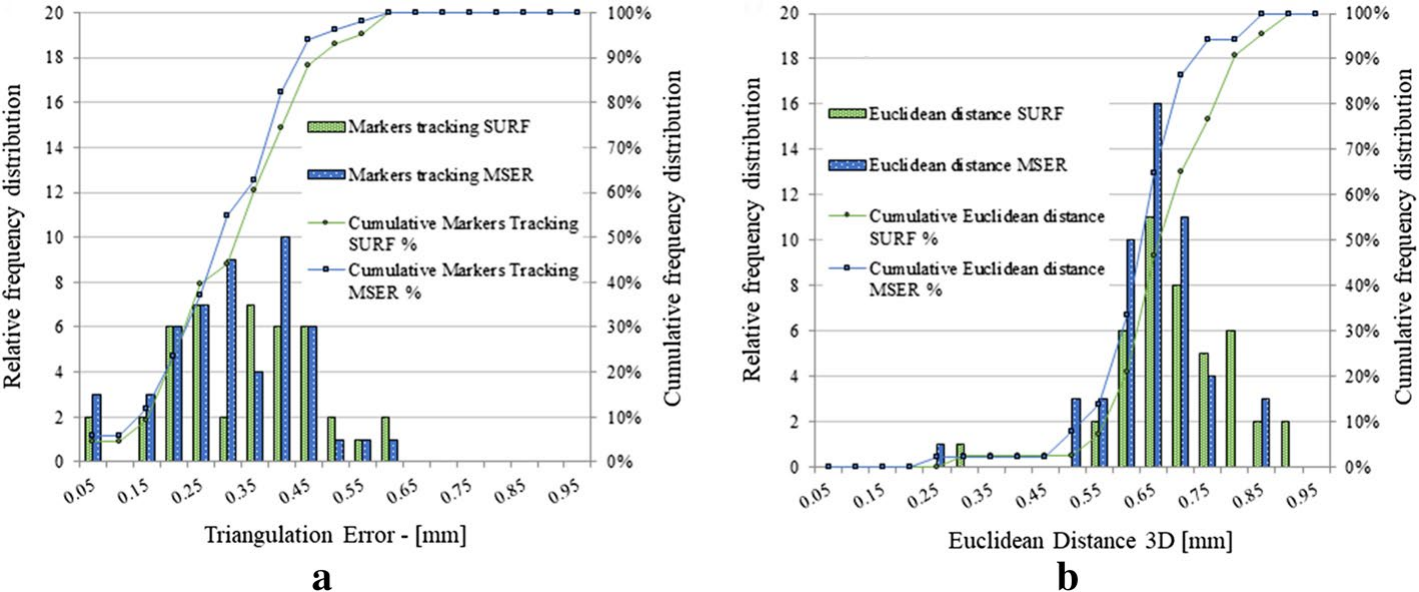
**a** Triangulation error and **b** Euclidean distance of detected optical markers for ground-truth comparison

The marker detection and ground-truth comparisons were performed by applying both MSER and SURF feature detection algorithms, to detect the optical markers positioned on the patient. The mean triangulation error of the tracked markers was 0.290 mm for MSER and 0.303 mm for SURF, as shown in Table 4. Table 4 portrays that an average Euclidean distance of 0.627 mm for MSER and that of 0.622 mm for SURF are reached, in relation to the ground truth. Descriptive statistics for triangulation error and Euclidean distance when the detection methods are applied to the optical markers are reported in Table 4. Figure 6a, b, shows the frequency distributions of triangulation errors and Euclidean distances for MSER and SURF detection methods. Notably, the thresholding performed for segmenting the optical markers prior to applying the feature detection can cause a non-ideal identification of the markers and decrease the triangulation accuracy. This is the main reason why the coordinates of the triangulated markers differ slightly with respect to the ground truth. However, all markers are triangulated with a sub-millimeter accuracy, resulting in a triangulation error comparable to the one obtained with the skin features.

**Table 4:** Optical marker comparison, triangulation error, and Euclidian distance

Optical marker detection	Triangulation error		Euclidean distance	
Method	MSER	SURF	MSER	SURF
Markers amount	52	43	52	43
Mean [mm]	0.290	0.303	0.627	0.622
Standard deviation [mm]	0.124	0.133	0.101	0.107
Rms [mm]	0.315	0.331	0.635	0.631
Min [mm]	0.018	0.028	0.232	0.223
Quartile 1 [mm]	0.207	0.202	0.579	0.567
Median [mm]	0.279	0.313	0.621	0.624
Quartile 3 [mm]	0.390	0.399	0.685	0.684
Max [mm]	0.557	0.595	0.840	0.823

## Discussion

This feasibility study proposes a new innovative, accurate, and unobtrusive alternative approach for skin feature localization which can be used for patient tracking in surgical navigation. This result was achieved with the direct detection of features on the patient’s skin using high-resolution grey-scale cameras and the subsequent analysis of the captured multi-view images. The framework is based on MSER and SURF feature detection algorithms to localize the visual skin features.

The accuracy, of roughly 0.6 mm at skin level, achieved with the current framework should be seen in light of previous results obtained by the ARSN system relying on adhesive skin markers. In a recent study using ARSN, Burström et al. [32] demonstrated a technical accuracy of 0.94 ± 0.59 mm and 1.97 ± 1.33 mm, respectively, for cadaveric and clinical cases of pedicle screw placement. The difference in accuracy, could be attributed to the actual placement of pedicle screws in a clinical situation. Studies on markerless tracking in surgery are scarce. In the early 2000, a series of articles regarding augmented reality for surgical planning using video projectors were published [33–38]. An off the shelf video camera and a 3D surface scanner were used to create a representation of the surface of the patient. Previously segmented structures could then be projected back on the patient. This system made dynamic tracking of the patient possible and reached a high accuracy of 1.5 mm. It has been used for radiotherapy and in craniofacial surgery, but no use in spine has ever been published [39]. Microsoft Hololens has recently been used in many applications. The obtained accuracies range from 9 to 45 mm in non-medical to roughly 5 mm in a spine phantom study [14, 15]. A recent robotic study using a structured light camera for markerless tracking of the bony anatomy reached a precision of 0.28 ± 0.16 mm [13]. A study using a similar approach as used here for spine feature tracking reached an accuracy of 0.5 mm [31]. When exploring the use of HSI to detect skin features in 2D on healthy volunteers, an accuracy better than 0.25 mm was achieved [40]. In the current study, the feasibility of 3D-localization of skin features in patients undergoing spine surgery was demonstrated, employing a pre-existing surgical navigation system using optical cameras. The use of grey-scale cameras rather than HSI is motivated by several factors. First, HSI is highly dependent on proper lighting conditions. Surgical lights illuminating the skin surface can interfere with the image acquisition process [40]. Second, the HSI system did not reach deeper than 1 mm in the skin, limiting its added value. Third, the integration of two or more HS cameras in the navigation system, to enable stereo-vision, would come at a considerable cost. In this scenario, the use of grey-scale cameras represents a good comprise.

The results obtained by the proposed framework, using grey-scale cameras, are thus well in line with these previous results. A markerless tracking framework has the advantage of building a virtual reference grid that cannot be dislodged or completely obscured during surgery. Furthermore, compared to conventional dynamic reference frames that track a single vertebra, a markerless framework can track the entire spine and compensate for inherent movements within the spinal column during surgery. In this study, several datasets were used for validation. MSER showed a better capability of detecting features of observable anatomical skin details. It was visually verified that MSER provided a higher number of detected features contributing to a better plane selection in the 3D outlier removal step. The multi-camera system enabled triangulation of each feature, to obtain an accurate 3D triangulation performance. This performance can be potentially evaluated by automatically computing the triangulation error continuously and in real time within a software function for navigation. The proposed framework may simplify the existing patient preparation procedure and improve the reliability of the tracking process by relying on skin features instead of optical markers or reference frames.

## Limitations

The limitation of this study is the small sample size and the retrospective setup. The results were validated on eight clinical cases. The addition of more cases would strengthen the conclusions. A prospective study comparing different modes of patient tracking (different types of optical markers versus different frameworks for feature tracking) is warranted. Another important issue is to reach enough computational power for real-time tracking of movements and surgical navigation.

## Conclusion

This study demonstrates the feasibility of skin feature localization by exploiting an optical multi-view, grey-scale, camera system combined with image analysis and tracking techniques. The system has been tested on several patients undergoing spinal surgery with sub-millimeter accuracy. This study can be the basis for future surgical applications where optical patient tracking is required.

## Methods

### Image preprocessing

The principles of multi-view geometry [41] are based on assuming a pinhole camera model, applied for correction of camera images with respect to intrinsic parameters. A preprocessing step, the Contrast-Limited Adaptive Histogram Equalization (CLAHE) algorithm, was used to maximize the detection of skin features and reduce the noise during the acquisition [42]. The simple computation of the fundamental matrix F with the normalized eight-point algorithm was used for image rectification [43]. The fundamental matrix *F* was defined as:

1 $$\begin{aligned} (x')^T\cdot F\cdot x = 0. \end{aligned}$$

For any pair of matching points $$ x'$$ and *x*, there are two images in the same coordinate system. The obtained pixel points were imposed on the corrected input images, to enable feature detection.

### Feature detection, extraction, and matching

Let us consider an image pair captured with different cameras $c_i$ and $c_j$, from different views: $Ic_i$ and $Ic_j$. For both images, a corresponding set of $n(c_i )$ and $n(c_j )$ features, respectively, were extracted and saved in a dedicated object ensemble $Fc_i$ and $Fc_j$ , to capture all information of the detected features *f*(*c*, *n*):

2 $$\begin{aligned} Fc_i= & {} f(c_i,1), f(c_i,2), . . .,f(c_i,n(c_i ) ), \end{aligned}$$

3 $$\begin{aligned} Fc_j= & {} f(c_j,1),f(c_j,2),. . .,f(c_j,n(c_j ) ). \end{aligned}$$

MSER and SURF algorithms were applied for blob-similar feature design and feature detection. Afterward, the SURF feature descriptor was applied for feature extraction. The regions of interest (ROIs) on the skin were selected manually and saved as bounding-box coordinates for future iterations. A manual selection was performed, because the regions of the same subject were located within different views. Unfortunately, it was not possible to manually select precisely the same region boundary within multiple views. For this reason, the matching process contained some outliers, which were filtered out at a later step of the processing. Attention was paid to select regions where the skin was as flat as possible. The chosen skin area was located around the optical markers (used as ground truth), so that both the markers and skin area had the same illumination conditions. This manual extraction may represent a limitation for a real-time application. For every detected feature, it was necessary to extract a feature vector, known as descriptor that provided information about the feature, in this case the pixels surrounding the center of the blob. For this purpose, the SURF algorithm was used to extract the feature vector [27]. This method was adopted, since it offers high reproducibility, even under different viewing conditions. The descriptor vectors were then saved in the following set of dedicated descriptor vectors:

4 $$\begin{aligned} \Delta _ci= & {} \delta (c_i,1),\delta (c_i,2),...,\delta (c_i,n(c_i ) ), \end{aligned}$$

5 $$\begin{aligned} \Delta _cj= & {} \delta (c_j,1),\delta (c_j,2),...,\delta (c_j,n(c_j ) ). \end{aligned}$$

Where every descriptor $\delta (c,n)$ consisted of an SURF descriptor vector and the y-coordinate of a specific feature *n* from a generic camera *c*. Using the previous dedicated descriptor vectors $\Delta _ci$ and $\Delta _cj$, the feature matching step performed a matching between the $(c_i)$ feature detected in one view with respect to the $n(c_j)$ feature from another view and provided an index of correspondences between the two dedicated descriptor vectors $\Delta _ci$ and $\Delta _cj$, and the feature vectors $F_cj$ and $F_cj$. These correspondences were achieved by computing the SSD between the SURF descriptor vectors of those features lying within the scan lines of interest. At this point, fusing the epipolar constraint was crucial, since it leads the matching process between features that were shifted along an epipolar line for a specific range. Thanks to this top–bottom scan-line stereo matching, matches between features that lie on different epipolar lines were omitted, to reduce the computational cost and maximize the chance of a good match. This step returned two vectors with the indexes related to the matched features. Using these indexes, it was possible to build two new dedicated object ensembles $M_ci$ and $M_cj$ of equal size, with matched features.

### Feature triangulation and outlier removal

**Fig. 7:**
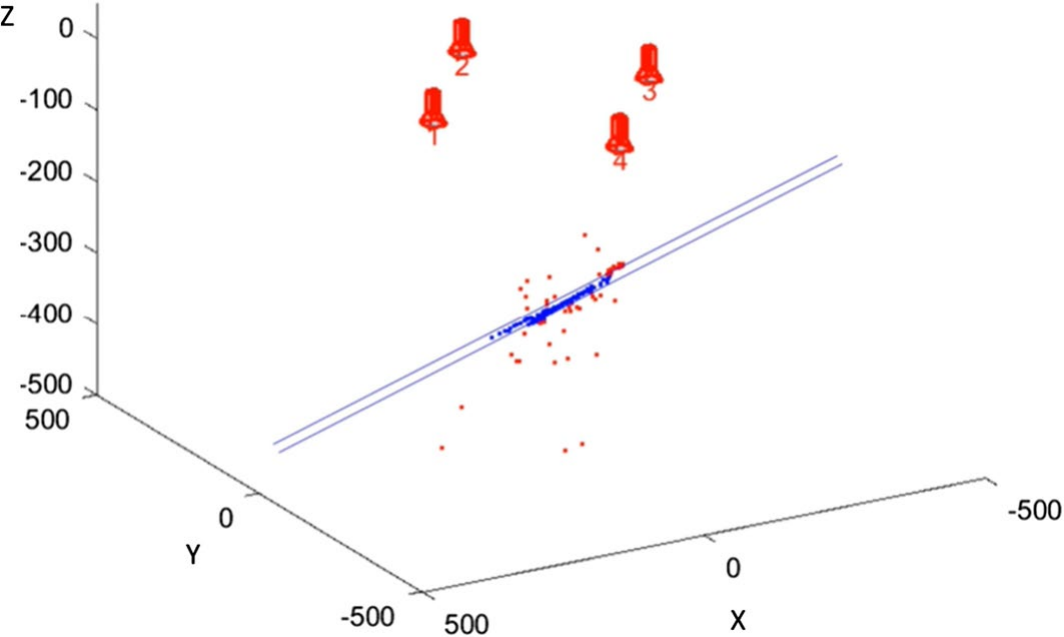
3D outlier removal: the 3D point cloud in blue represents the inliers, while in red, the 3D point cloud depicts the outliers which are the discarded keypoints. On the top, the numerated cameras

In computer vision, triangulation allows determination of the 3D position of a point, given that the positions of the same points are matched in at least two alternative views [41]. This was achieved with two or more lines projected from each camera center to the respective point on the camera plane. Consequently, the projected lines did not always intersect in the same 3D point. It was important to evaluate and quantify the accuracy of this method. In this feasibility study, the triangulation error was used as evaluation metric to obtain an index of the triangulation accuracy, and then, the 3D point locations were used to perform a benchmark against an existing tracking system. The triangulation error was computed by calculating the location of the shortest distance between the two projected lines. The center of the line segment was the triangulated point, and the length of the line segment is the triangulation error, expressed in millimeters (or in micrometers). The triangulation function of the ARSN system returns the 3D Cartesian coordinates of the triangulated point and the corresponding triangulation error through the projections from the camera centers, resulting in:

6 $$\begin{aligned} \Psi (c_i|c_j,n) = x((c_i|c_j),n),y (c_i|c_j ,n),z(c_i|c_j,n),e (c_i|c_j,n)), \end{aligned}$$

where $$ c_i|c_j $$ denotes the pair of cameras used for the triangulation, $$x(c_i|c_j ,n)$$, $$y(c_i|c_j,n)$$, $$z(c_i|c_j,n)$$ are the 3D Cartesian coordinates of the triangulated feature *n*, and $$e(c_i|c_j,n)$$ is the triangulation error of the triangulated feature *n* with cameras $$c_i|c_j$$. These 3D coordinates were stored in a dedicated object vector $$\Psi c_i|c_j$$ for further analysis. The triangulation was achievable between at least two camera pairs. In fact, it was possible to obtain the rectification for the six camera pairs: $$c_i|c_j =c_1|c_2$$, $$c_1|c_3 $$, $$c_1|c_4 $$, $$c_2|c_3$$, $$c_2|c_4 $$, and $$c_3|c_4 $$, and then detect, match, and triangulate the features with at least one of these pairs, mainly determined by choosing a more favorable line-of-sight that avoids occlusion of the analyzed regions. The overall mean triangulation error was computed by averaging the triangulation errors of all the features detected over multiple regions. At this point, most of the matches were obtained by only relying on the epipolar constraint. Using the outlier removal step a 3D constraint was imposed, by approximating the skin surface to a simple planar representation, in which all the detected features were situated. Now, M-estimator SAmple and Consensus (MSAC) [44] was used to fit a plane to the 3D point cloud and remove triangulated points that lie below or above a certain maximum distance from the plane model (expressed in mm). The reference orientation constraint was inferred from the mechanical parameters of the C-arm once the position of the multi-view camera system with respect to the skin surface was obtained. It was important to quantify the considered region of interest for algorithm comparison and the applicability of the approach. For this purpose, a simple method for skin-area estimation was applied by considering the triangulated features included in the approximated skin-model plane. A flat mesh plot was created from the coordinates obtained by intersecting the normal projections of the features onto the model plane. The overall area was computed as the sum of all the Delaunay triangulation areas included within the boundaries of the region [45]. The size of the analyzed region was then expressed in square centimeters. Figure 7 shows the 3D point cloud that represents the inliers (in blue), and the 3D point cloud depicting the outliers (in red), which are the discarded features. Both are reconstructed from the four high-resolution optical cameras illustrated on top.

### Marker localization and ground-truth comparison

**Fig. 8:**
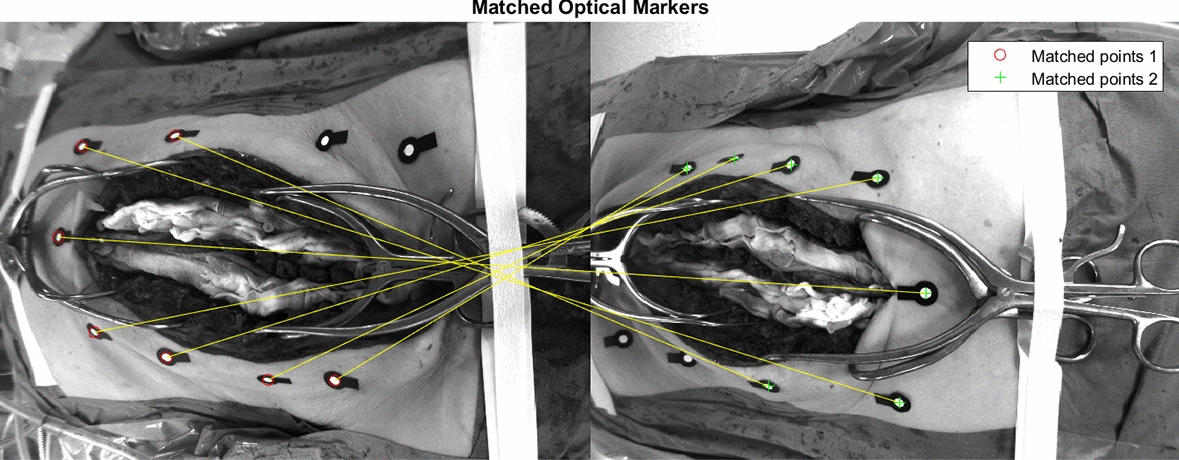
Matching of the optical markers

The accuracy of the proposed framework was evaluated by detection 3D triangulation of the optical markers, which were considered as ground truth. The detection and matching of the optical markers were performed in two different views of the patients. For this purpose, a preprocessing step consisting of a segmentation mask and a simple binarization thresholding was applied. At the end, the MSER and SURF methods were used to perform the feature detection of the bright circular region of every optical marker. The final matching process for a pair of images is shown in Fig. 8. When the marker matching was performed, their triangulation was computed, and their 3D location was obtained. The benchmarking was performed with respect to the ground truth of each optical marker provided by the ARSN system. The Euclidean distances in 3D between the detected marker and the corresponding ground-truth marker coordinates were computed to assess the accuracy of the localization.

## Data Availability

Restrictions apply to the availability of these data, which were used under license within a European project (see Acknowledgements) for the current study, and so are not publicly available.

## References

[1] Yoon JW, Wang MY (2019). The evolution of minimally invasive spine surgery: Jnspg 75th anniversary invited review article. J Neurosurg Spine.

[2] Mobbs RJ, Sivabalan P, Li J (2011). Technique, challenges and indications for percutaneous pedicle screw fixation. J Clin Neurosci.

[3] Devito DP, Kaplan L, Dietl R, Pfeiffer M, Horne D, Silberstein B, Hardenbrook M, Kiriyanthan G, Barzilay Y, Bruskin A (2010). Clinical acceptance and accuracy assessment of spinal implants guided with spineassist surgical robot: retrospective study. Spine.

[4] Burström G, Nachabe R, Persson O, Edström E, Terander AE (2019). Augmented and virtual reality instrument tracking for minimally invasive spine surgery: a feasibility and accuracy study. Spine.

[5] Hott JS, Deshmukh VR, Klopfenstein JD, Sonntag VK, Dickman CA, Spetzler RF, Papadopoulos SM (2004). Intraoperative iso-c c-arm navigation in craniospinal surgery: the first 60 cases. Neurosurgery.

[6] Elmi-Terander A, Nachabe R, Skulason H, Pedersen K, Söderman M, Racadio J, Babic D, Gerdhem P, Edström E (2018). Feasibility and accuracy of thoracolumbar minimally invasive pedicle screw placement with augmented reality navigation technology. Spine.

[7] Elmi-Terander A, Burström G, Nachabé R, Fagerlund M, Ståhl F, Charalampidis A, Edström E, Gerdhem P (2020). Augmented reality navigation with intraoperative 3d imaging vs fluoroscopy-assisted free-hand surgery for spine fixation surgery: a matched-control study comparing accuracy. Sci Rep.

[8] Van de Kelft E, Costa F, Van der Planken D, Schils F (2012). A prospective multicenter registry on the accuracy of pedicle screw placement in the thoracic, lumbar, and sacral levels with the use of the o-arm imaging system and stealthstation navigation. Spine.

[9] Malham GM, Parker RM (2018). Early experience of placing image-guided minimally invasive pedicle screws without k-wires or bone-anchored trackers. J Neurosurg Spine.

[10] Virk S, Qureshi S (2019). Navigation in minimally invasive spine surgery. J Spine Surg.

[11] Suenaga H, Tran HH, Liao H, Masamune K, Dohi T, Hoshi K, Takato T (2015). Vision-based markerless registration using stereo vision and an augmented reality surgical navigation system: a pilot study. BMC Med Imag.

[12] Seitel A, Bellemann N, Hafezi M, Franz AM, Servatius M, Saffari A, Kilgus T, Schlemmer H-P, Mehrabi A, Radeleff BA (2016). Towards markerless navigation for percutaneous needle insertions. Int J Comput Assist Radiol Surg.

[13] Zhu S, Zhao Z, Pan Y, Zheng G (2020). Markerless robotic pedicle screw placement based on structured light tracking. Int J Comput Assist Radiol Surg.

[14] Hübner P, Clintworth K, Liu Q, Weinmann M, Wursthorn S (2020). Evaluation of hololens tracking and depth sensing for indoor mapping applications. Sensors.

[15] Gibby JT, Swenson SA, Cvetko S, Rao R, Javan R (2019). Head-mounted display augmented reality to guide pedicle screw placement utilizing computed tomography. Int J Comput Assist Radiol Surg.

[16] Edström E, Burström G, Nachabe R, Gerdhem P, Elmi Terander A (2020). A novel augmented-reality-based surgical navigation system for spine surgery in a hybrid operating room: design, workflow, and clinical applications. Operat Neurosurg.

[17] Elmi-Terander A, Burström G, Nachabe R, Skulason H, Pedersen K, Fagerlund M, Ståhl F, Charalampidis A, Söderman M, Holmin S (2019). Pedicle screw placement using augmented reality surgical navigation with intraoperative 3d imaging: a first in-human prospective cohort study. Spine.

[18] Dieterich S, Tang J, Rodgers J, Cleary K. Skin respiratory motion tracking for stereotactic radiosurgery using the cyberknife. In: International Congress Series; 2003, vol. 1256, p. 130–6. Elsevier.

[19] Helm PA, Teichman R, Hartmann SL, Simon D (2015). Spinal navigation and imaging: history, trends, and future. IEEE Trans Med Imag.

[20] Burström G, Buerger C, Hoppenbrouwers J, Nachabe R, Lorenz C, Babic D, Homan R, Racadio JM, Grass M, Persson O (2019). Machine learning for automated 3-dimensional segmentation of the spine and suggested placement of pedicle screws based on intraoperative cone-beam computer tomography. J Neurosurg Spine.

[21] Wang F, Behrooz A, Morris M (2013). High-contrast subcutaneous vein detection and localization using multispectral imaging. J Biomed Optics.

[22] Yang R, Wang Z, Liu S, Wu X (2012). Design of an accurate near infrared optical tracking system in surgical navigation. J Lightwave Technol.

[23] Asrar M, Al-Habaibeh A, Houda M (2016). Innovative algorithm to evaluate the capabilities of visual, near infrared, and infrared technologies for the detection of veins for intravenous cannulation. Appl Optics.

[24] Xue Y, Cheng T, Xu X, Gao Z, Li Q, Liu X, Wang X, Song R, Ju X, Zhang Q (2017). High-accuracy and real-time 3d positioning, tracking system for medical imaging applications based on 3d digital image correlation. Optics Lasers Eng.

[25] Donoser M, Riemenschneider H, Bischof H. Shape guided maximally stable extremal region (mser) tracking. In: 2010 20th international conference on pattern recognition; 2010, p. 1800–3. IEEE.

[26] Donoser M, Bischof H. Efficient maximally stable extremal region (mser) tracking. In: 2006 IEEE computer society conference on computer vision and pattern recognition (CVPR’06); 2006, vol. 1, p. 553–560. IEEE.

[27] Bay H, Tuytelaars T, Van Gool L. Surf: speeded up robust features. In: European conference on computer vision; 2006, p. 404–17. Springer.

[28] Manni F, Mamprin M, Zinger S, Shan C, Holthuizen R, de With P. Multispectral image analysis for patient tissue tracking during complex interventions. In: 2018 25th IEEE international conference on image processing (ICIP); 2018, p. 3149–53. IEEE.

[29] Moura GM, Da Silva RLDS (2017). Analysis and evaluation of feature detection and tracking techniques using open cv with focus on markerless augmented reality applications. J Mob Multimedia.

[30] Ta D-N, Chen W-C, Gelfand N, Pulli K. Surftrac: efficient tracking and continuous object recognition using local feature descriptors. In: 2009 IEEE conference on computer vision and pattern recognition; 2009, p. 2937–44. IEEE.

[31] Manni F, Elmi-Terander A, Burström G, Persson O, Edström E, Holthuizen R, Shan C, Zinger S, van der Sommen F (2020). Towards optical imaging for spine tracking without markers in navigated spine surgery. Sensors.

[32] Burström G, Nachabe R, Homan R, Hoppenbrouwers J, Holthuizen R, Persson O, Edström E, Elmi-Terander A (2020). Frameless patient tracking with adhesive optical skin markers for augmented reality surgical navigation in spine surgery. Spine.

[33] Hoppe H, Dauber S, Raczkowsky J, Worn H, Moctezuma JL. Intraoperative visualization of surgical planning data using video projectors. In: Studies in health technology and informatics; 2001, p. 206–8. 11317740

[34] Hoppe H, Däuber S, Kübler C, Raczkowsky J, Wörn H (2002). A new, accurate and easy to implement camera and video projector model. Stud Health Technol Inform.

[35] Dauber S, Hoppe H, Krempien R, Hassfeld S, Brief J, Worn H. Intraoperative guidance of pre-planned bone deformations with a surface scanning system. In: Studies in health technology and informatics; 2002, p. 110–5. 15458069

[36] Eggers G, Salb T, Hoppe H, Kahrs L, Ghanai S, Sudra G, Raczkowsky J, Dillmann R, Worn H, Hassfeld S (2005). Intraoperative augmented reality: the surgeons view. Stud Health Technol Inform.

[37] Kahrs LA, Hoppe H, Eggers G, Raczkowsky J, Marmulla R, Wörn H (2005). Visualization of surgical 3d information with projector-based augmented reality. Stud Health Technol Inform.

[38] Marmulla R, Hoppe H, Mühling J, Eggers G (2005). An augmented reality system for image-guided surgery: this article is derived from a previous article published in the journal international congress series. Int J Oral Maxillofacial Surg.

[39] Wörn H, Aschke M, Kahrs LA (2005). New augmented reality and robotic based methods for head-surgery. Int J Med Robot Comput Assist Surg.

[40] Manni F, van der Sommen F, Zinger S, Shang C, Holthuizen R, Lai M, Buström G, Hoveling RJ, Edström E, Elmi-Terander A (2020). Hyperspectral imaging for skin feature detection: Advances in markerless tracking for spine surgery. Appl Sci.

[41] Multiple View Geometry in Computer Vision. Cambridge university press

[42] Zuiderveld K. Contrast limited adaptive histogram equalization. Graphics gems; 1994, p.474–85.

[43] Hartley RI (1997). In defense of the eight-point algorithm. IEEE Trans Pattern Anal Mach Intell.

[44] Torr PH, Zisserman A (2000). Mlesac: a new robust estimator with application to estimating image geometry. Comput Vis Image Understanding.

[45] Aurenhammer F, Klein R, Lee D-T. Voronoi diagrams and delaunay triangulations; 2013.

